# Prognostic Significance of Amino Acid and Biogenic Amines Profiling in Chronic Kidney Disease

**DOI:** 10.3390/biomedicines11102775

**Published:** 2023-10-13

**Authors:** Guillermo Gervasini, Zoraida Verde, Luz M. González, Celia Chicharro, Laura González-Rodríguez, Ana Fernández-Araque, Sonia Mota-Zamorano, Bárbara Cancho, Alberto Pérez-Hernández, Virginio García-López, Fernando Bandrés, Nicolás R. Robles

**Affiliations:** 1 Department of Medical and Surgical Therapeutics, Medical School, University of Extremadura, 06006 Badajoz, Spain; lmgonzalezgarcia89@gmail.com (L.M.G.); lgonzrodrig@gmail.com (L.G.-R.); smotazamorano@gmail.com (S.M.-Z.); garcialopez@unex.es (V.G.-L.); 2 Institute of Molecular Pathology Biomarkers, University of Extremadura, 06006 Badajoz, Spain; 3 RICORS2040 Renal Research Network, 28029 Madrid, Spain; nrrobles@unex.es; 4 Department of Biochemistry, Molecular Biology and Physiology, Universidad de Valladolid, 42005 Soria, Spain; zoraida.verde@uva.es; 5 GIR—Pharmacogenetics, Cancer Genetics, Genetic Polymorphisms and Pharmacoepidemiology, University of Valladolid, 47005 Valladolid, Spain; anamaria.fernandez@uva.es; 6 Research Group Centro de Estudios Gregorio Marañón, Fundación Ortega-Marañón, 28010 Madrid, Spain; celchich@ucm.es (C.C.); fbandres@med.ucm.es (F.B.); 7 Biopathology-Toxicology Laboratory, Department of Legal Medicine, Psychiatry and Pathology, Faculty of Medicine, University Complutense of Madrid, 28040 Madrid, Spain; 8 Department of Nursery, University of Valladolid, 42005 Soria, Spain; 9 Service of Nephrology, Badajoz University Hospital, 06006 Badajoz, Spain; bcancho@yahoo.es; 10 Department of Clinical Biochemistry, Hospital Santa Bárbara, 42005 Soria, Spain; aperezhe@saludcastillayleon.es

**Keywords:** chronic kidney disease, end-stage kidney disease, amino acids, metabolites

## Abstract

There is a pressing need for more precise biomarkers of chronic kidney disease (CKD). Plasma samples from 820 subjects [231 with CKD, 325 with end-stage kidney disease (ESKD) and 264 controls] were analyzed by liquid chromatography with tandem mass spectrometry (LC-MS/MS) to determine a metabolic profile of 28 amino acids (AAs) and biogenic amines to test their value as markers of CKD risk and progression. The kynurenine/tryptophan ratio showed the strongest correlation with estimated glomerular filtration rate values (coefficient = −0.731, *p* < 0.0001). Models created with orthogonal partial least squares-discriminant analysis (OPLS-DA) containing the metabolic signature showed a high goodness of fit and predictability for controls/CKD (R2X:0.73:R2Y:0.92:Q2:0.92, *p* < 0.0001) and lower values for CKD/ESKD (R2X:0.56:R2Y:0.59:Q2:0.55, *p* < 0.0001). Based on generated VIP scores, the most relevant markers for segregating samples into control/CKD and CKD/ESKD groups were citrulline (1.63) and tryptophan (1.47), respectively. ROC analysis showed that the addition of the metabolic profile to a model including CKD classic risk factors improved the AUC from 86.7% (83.6–89.9) to 100% (100–100) for CKD risk (*p* < 0.0001) and from 63.0% (58.2–67.8) to 96.5% (95.3–97.8) for the risk of progression from CKD to ESKD (*p* < 0.0001). Plasma concentrations of AAs and related amines may be useful as diagnostic biomarkers of kidney disease, both for CKD risk and for progression of CKD patients to ESKD.

## 1. Introduction

Chronic kidney disease (CKD), defined by abnormalities of renal function and/or structure over 3 months with clinical consequences, is a global healthcare issue that has been predicted to become one of the leading global causes of death by 2040 [1]. Furthermore, its prevalence has significantly increased in the last decades, so much so that up to 10–15% of adults worldwide are affected. The main burdens of CKD are not, as it is perceived by many, dialysis and kidney transplant; rather, accelerated aging and premature death, often associated with increased cardiovascular risk, are the most prominent concerns [2].

One specific aspect of CKD, particularly in early stages, is that often times patients do not show overt clinical symptoms; therefore, laboratory tests are crucial to monitor the disease. In this regard, the assessment of glomerular filtration rate (GFR) is key to evaluate renal function and is usually estimated (eGFR) based on serum creatinine concentrations using different equations, of which CKD-EPI is presently recommended. However, creatinine levels begin to stand out only when the disease is well under way and have been shown to be less accurate under special circumstances. In addition, eGFR is of little help in assessing the risk of progression to end-stage kidney disease (ESKD). In fact, even though CKD prevalence based on eGFR measurements is very similar in Western countries, the incidence of patients requiring renal replacement therapy is very different among them [3]. Available clinical evidence strongly indicates that patients could greatly benefit from novel biomarkers that are able to identify patients at risk at an early stage.

Renal function affects the levels of metabolites in blood; hence, it is likely that some metabolites could be helpful in estimating kidney function. In this regard, the kidney plays a crucial role in the metabolism of proteins and amino acids (AAs), which are constantly filtered and reabsorbed [4]. Quantitative analysis of free AAs in blood and urine has been shown useful for the diagnosis and management of metabolic disturbances as well as for the assessment of nutritional status, tissue damage and renal function [5]. In this regard, mass spectrometry (MS)-based methods have been applied to the study of CKD with great success [6]. The development of nondestructive methods for protein ionization was the critical milestone that allowed the determination of biomolecules with MS. Electrospray ionization (ESI), which is used in the present study, is one of such methods that is compatible with proteins dissolved at typical concentration ranges of 10^−6^ to 10^−15^ M [7]. As described in the Section 2, the methodology used for this study was based on liquid chromatography coupled with tandem mass spectrometry (LC-MS/MS). Early studies focused on the signal response of proteins in complex mixtures have shown a linear correlation of protein concentrations with MS-MS spectral values [8]. These studies have set the framework for efficient quantitative analysis of LC-MS/MS such as that presented herein.

The advent of metabolomics, which identifies and quantitatively compares small-molecule metabolites between different groups, has allowed the identification of several circulating AAs and related compounds that may be associated with CKD [9,10,11]. However, results are mostly based on small cohorts of renal patients, and oftentimes a comparison between different stages of the disease is lacking.

The goal of this work was to determine plasma concentrations of AAs and biogenic amines in a large group of healthy subjects, CKD patients, and patients with ESKD to identify differences in their metabolic profiles and analytes with the potential to become biomarkers of renal disease.

## 2. Materials and Methods

### 2.1. Subjects

Between 2017 and 2022, 820 Caucasian Spanish subjects were recruited, namely (i) 231 patients diagnosed with different CKD stages [45 (19.5%) with stage 1–2, 69 (29.9%) with stage 3, and 117 (50.6%) with stage 4] at the Nephrology Service of the Badajoz University Hospital (Spain); (ii) 325 patients with end-stage kidney disease (ESKD, eGFR < 15 mL/min/1.73 m^2^), who were enrolled at the Advanced CKD Unit of the Badajoz University Hospital and at three Dialysis Units (Fresenius clinic and Llerena and Zafra hospitals in Badajoz, Spain); and finally, (iii) 264 healthy volunteers who were recruited from primary care clinics in Soria, Spain. Transplantation, pregnancy or breastfeeding, active infection, cancer, or acute kidney injury were all considered exclusion criteria. All patients were over 18 years of age and gave written consent for their participation in the study, which was approved by the Ethics Committees of the Badajoz University Hospital (21 April 2022) and was carried out in accordance with the Declaration of Helsinki and its subsequent revisions.

Diagnostic and prognostic stratification of renal patients was conducted with the KDIGO classification and table of progression risk and the CONSORTIUM-CKD equation. Kidney function was assessed using the CKD-EPI equation. Clinical records were examined to retrieve clinical, demographic, and biochemical data.

### 2.2. Sample Collection and Amines Analyzed

Blood samples (3 mL) were drawn in EDTA tubes from each participant, and plasma was immediately obtained by centrifuging 10 min at 3000 rpm. Plasma samples were then aliquoted (500 μL) and stored at −80 °C until analysis. Essential AA (EAAs), including histidine, isoleucine, leucine, lysine, methionine, phenylalanine, threonine, tryptophan, and valine, and non-essential AA (NEAAs), including alanine, arginine, asparagine, aspartic acid, citrulline, glutamic acid, glutamine, glycine, proline, L-serine, and tyrosine, were determined. In addition, eight other related metabolites and biogenic amines, namely kynurenic acid, kynurenine, asymmetric dimethyl arginine (ADMA), D-serine, serotonin, gamma aminobutyric acid (GABA), acetylcholine, and creatine, were also analyzed.

Ten different ratios involving AAs were calculated as described by Duranton et al. [12]. EAA/NEAA, Val/Gly, and alanine/branched-chain amino acid (BCAA) ratios are used to assess the overall nutritional state; the kidney’s ability for interconversions of AAs is measured using Tyr/Phe, Ser/Gly, and Arg/Cit ratios. The relative importance of both the kynurenine pathway and ADMA was assessed with the kynurenine/tryptophan ratio and the Arg/ADMA ratio. Finally, the Fisher’s ratio (defined as the sum of BCAAs, Leu, Ile, and Val divided by the sum of aromatic AAs, Tyr, and Phe) and the GSG index [Glu/(Ser + Gly)] were also calculated, as they have been observed to be altered in several pathologies and metabolic dysfunctions [13,14].

### 2.3. Chemical and Reagents

All chemical reagents were purchased from Sigma Aldrich (St. Louis, MO, USA). The water, methanol, and acetonitrile (J.T. Baker, Innovagen SL, Madrid, Spain) used for the mobile phases were all LC-MS grade. The stock solutions of asparagine, aspartate, glycine, and serine (300 μM); alanine, citrulline, glutamate, glutamine, histidine, kynurenic acid, kynurenine, methionine, phenylalanine, proline, threonine, tryptophan, and valine (50 μM); GABA, arginine, ADMA, leucine, isoleucine, lysine, serotonin, tyrosine, and acetylcholine (5 μM); and 20 mM isotopically labeled d4-acetylcholine were prepared in LCMS grade water (0.2 μm microfilter and bottled under nitrogen atmosphere). Then, a standard mixture was diluted from stocks with artificial cerebrospinal fluid, which consisted of 145 mM NaCl, 2.68 mM KCl, 1.4 mM CaCl_2_, 1.0 MgSO, 1.55 mM Na_2_HPO_4_, and 0.45 mM NaH_2_PO_4_ pH 7.4 at −80 °C (adjusted with NaOH).

### 2.4. Sample Preparation and LC/MS/MS Analysis

The procedure to prepare the samples and the calibration standards, listed in Supplemental Table S1, was the same, except that the samples started with a volume of 20 μL and the calibration standard with 5 μL. Cold acetonitrile (1:4) was added to precipitate proteins, and, with no need for incubation, samples were immediately centrifuged at 13,000 rpm for 10 min. Subsequently, 20 mL of the supernatant was derivatized by the sequential addition at room temperature of 10 μL of 100 mM Na_2_CO_3_, 10 μL of BzCl (2% *v*/*v* in acetonitrile), and 10 μL of the corresponding internal standard, as described by Wong et al. [15]. To balance the concentration of organic content, 50 μL of water was added. The calibration standards and the internal standard were frozen at −20 °C in aliquots to prevent multiple freeze/thaw cycles. An aliquot of the internal standard was thawed on the day of use, and a fresh benzoyl chloride (BzCl) solution was made daily.

**Figure 1:**
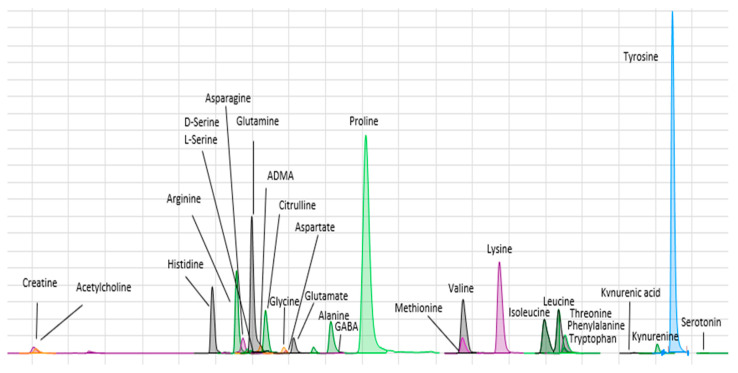
Chromatogram showing retention times for the amines assayed.

Amines were analyzed using LC/MS/MS. The technique was based on the method described by Wong et al. [15]. BzCl-derivatized samples were analyzed with LC-MS/MS using multiple reaction monitoring (MRM) dynamics with an Agilent 6410 TQ equipment. MRM conditions for all the metabolites are shown in Supplemental Table S2. Briefly, 5 μL of sample (by triplicate) was injected into a HiP-ALS automatic injection module at room temperature. An ACE Excel 2 SuperC18 column (15 cm × 2.1 mm ID, 2 μm, 90 Å) kept at 27 °C was used for the separation of metabolites. Mobile phase A consisted of 10 mM 0.15% formic acid ammonium formate, and mobile phase B was acetonitrile. The flow was set to 0.2 μL/min. The elution gradient was as follows: 5%; 0.0 min, 15% B; 0.01 min, 17% B; 0.5 min, 55% B; 14 min, 70% B; 14.5 min, 100% B; 18 min, 100% B; 19 min, 5% B; 19.1 min, 5% B; 24 min. An additional 10 min period of column equilibration at 0% B was required to achieve reproducible chromatography. The required pressure over the gradient ranged from 117 to 254 bar. ESI was used in positive mode at 4000 V. The gas temperature and flow were 350 °C and 11 L/min, respectively, with the nebulizer set to 15 psi. Automated peak integration was performed using Agilent MassHunter Quantitative Analysis for QQQ, version B.04.01. All peaks were visually inspected to ensure proper integration (Figure 1).

### 2.5. Statistical Analyses

Chi-square tests were utilized to compare categorical variables. Quantitative variables were compared between groups either using the *t*-test/Mann–Whitney test (2 groups) or ANOVA/Kruskal–Wallis test (>2 groups), depending on the normality of data distribution. Post-hoc tests were applied to examine differences between group pairs. Correlations between quantitative variables were assessed using the Spearman’s test. IBM SPSS v.22.0 (SPSS Inc., Chicago, IL, USA v.22.0) was utilized for the statistical analyses. Bonferroni correction for multiple testing was applied, resulting in a significance threshold of *p* < 0.0016.

In order to evaluate the impact of the different metabolic signatures, multivariate analyses were conducted with Umetrics SIMCA 18 software v. 18.0.0.372 (Umeå/Malmö, Sweden) to create orthogonal partial least squares-discriminant analysis (OPLS-DA) models, which were evaluated for fitness (R2) and predictive ability (Q2). This is a supervised dimension reduction analysis that allows visualization of how individuals within a group cluster with regard to their data-compressed principal components, i.e., it permits stratification of samples based on differences in profiles rather than individual variables. After removing outliers identified with principial component analysis (PCA), OPLS-DA models were then constructed based on 80% of the population and validated in the remaining 20% (training and test sets). These models were then utilized to identify the most significantly altered metabolites between groups by calculating the variable importance in the projection (VIP) score (values > 1 are considered relevant).

To assess the potential use of these metabolites as biomarkers to discriminate either between controls and CKD patients or between CKD and ESKD, areas under receiving operating characteristic (ROC) curves (AUCs) were calculated with R Studio for Windows v. 4.2.3 (pROC package). AUCs for models containing traditional risk factors before and after the addition of the metabolic signatures were compared using the De Long’s test.

## 3. Results

The study participants’ characteristics are summarized in Table 1.

The observed eGFR values ranged from 5.0 to 140.56 mL/min/1.73 m^2^. The percentage of males was higher in the CKD and ESKD group than in controls (*p* = 0.001). In addition, participants without renal impairment were older than CKD and ESKD patients (74.63 vs. 68.47 years, *p* < 0.0001). As expected, the incidence of diabetes mellitus (DM), hypertension and hyperlipidemia was much higher in CKD or ESKD patients than in controls (*p* < 0.0001 in all cases).

### 3.1. Quantification of Amino Acids and Amines in Plasma

Plasma concentrations (mmol/L) of the AAs and other amines analyzed are shown in Table 2. In univariate analyses and after Bonferroni correction for multiple testing, the differences among the three study groups were statistically significant for all the metabolites (*p* < 0.0001 in all cases). Post-hoc analyses were carried out to determine differences regarding metabolites levels between group pairs. Whilst differences between controls and CKD patients were also statistically significant for all metabolites, only 12 out of the 28 analytes assayed displayed significantly different levels between ESKD and CKD (Table 2). Next, multivariate linear regression models accounting for age, sex, BMI, diabetes, hypertension, smoking, and renal function were created to explain the observed metabolites concentrations. Supplemental Table S3 shows the resulting *p*-values and regression coefficients for the effect of renal status (control—CKD—ESKD) on the levels of each metabolite. Renal status was the main explanatory variable in all instances except for glutamine (beta coefficient = 0.073, *p* = 0.059), histidine (−0.283, *p* < 0.0001), acetylcholine (0.156, *p* < 0.0001) and kynurenine (−0.018, *p* = 0.755), for which diabetes had a higher regression coefficient.

### 3.2. Total Concentrations and Ratios of Amino Acids

**Figure 2:**
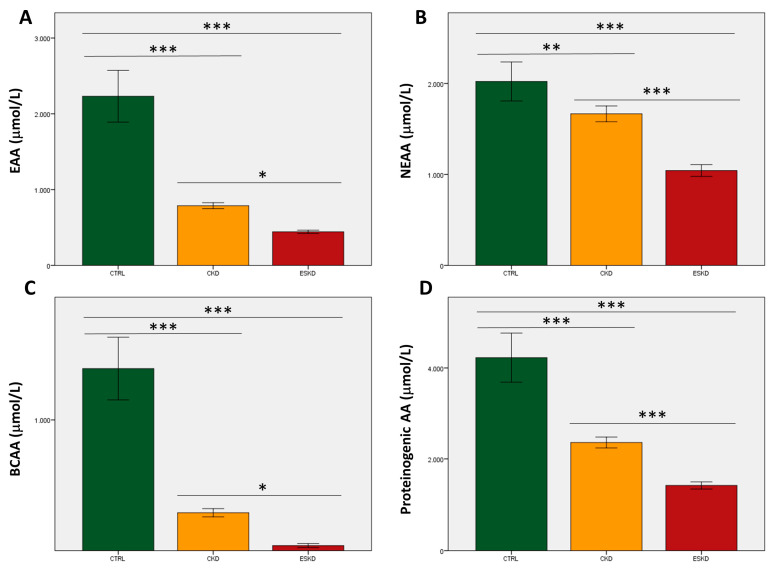
Total values of essential amino acids (**A**; EAAs), non-essential amino acids (**B**; NEAAs), branched-chain amino acids (**C**; BCAAs), and proteinogenic amino acids (**D**) in control subjects, chronic kidney disease (CKD) patients, and individuals with end-stage kidney disease (ESKD). * *p* < 0.05; ** *p* = 0.01; *** *p* < 0.0001.

**Figure 3:**
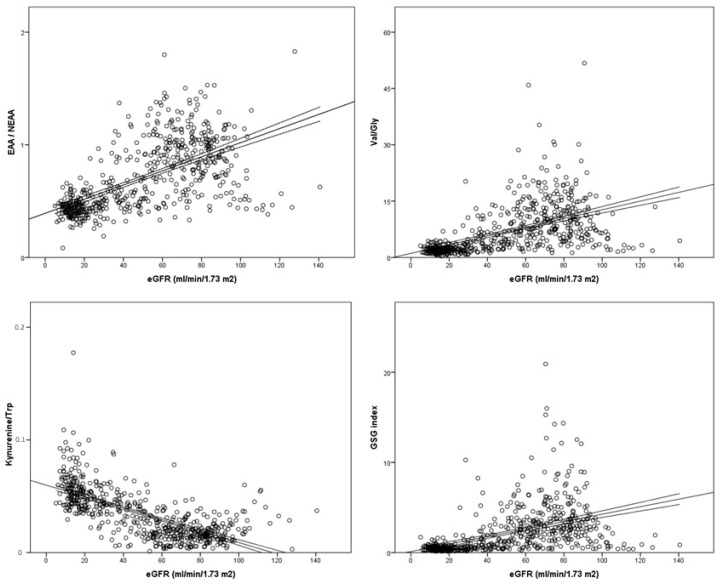
Plasma concentration of amino acid ratios in relation to estimated glomerular filtration rate (eGFR). Best-fit lines with 95% confidence intervals are shown.

With regard to total AA values, healthy subjects always displayed statistically significant higher plasma levels of EAAs, NEAAs, BCAAs and proteinogenic AAs than CKD patients, who in turn showed higher concentrations than ESKD patients (Figure 2). After correction for multiple testing, differences between the CKD and ESKD groups for both EAAs and BCAAs were reduced (*p* < 0.05; Figure 2A,C), whilst NEAAs and proteinogenic AAs maintained statistical significance (Figure 2B,D). We also analyzed 10 standard plasma AA ratios that were plotted against eGFR values to evaluate correlations in those participants who were not dialyzed. All correlations were significant (*p* < 0.0001). Three ratios displayed high coefficients between 0.65 and 0.70, namely EAA/NEAA (0.650), Val/Gly (0.676), and the GSG index (0.654), whilst the kynurenine/tryptophan ratio showed the strongest correlation with eGFR (coefficient = −0.731) (Figure 3). Data for the remaining six ratios are shown in Supplemental Figure S1.

### 3.3. Metabolic Profiles in the Study Groups

**Figure 4:**
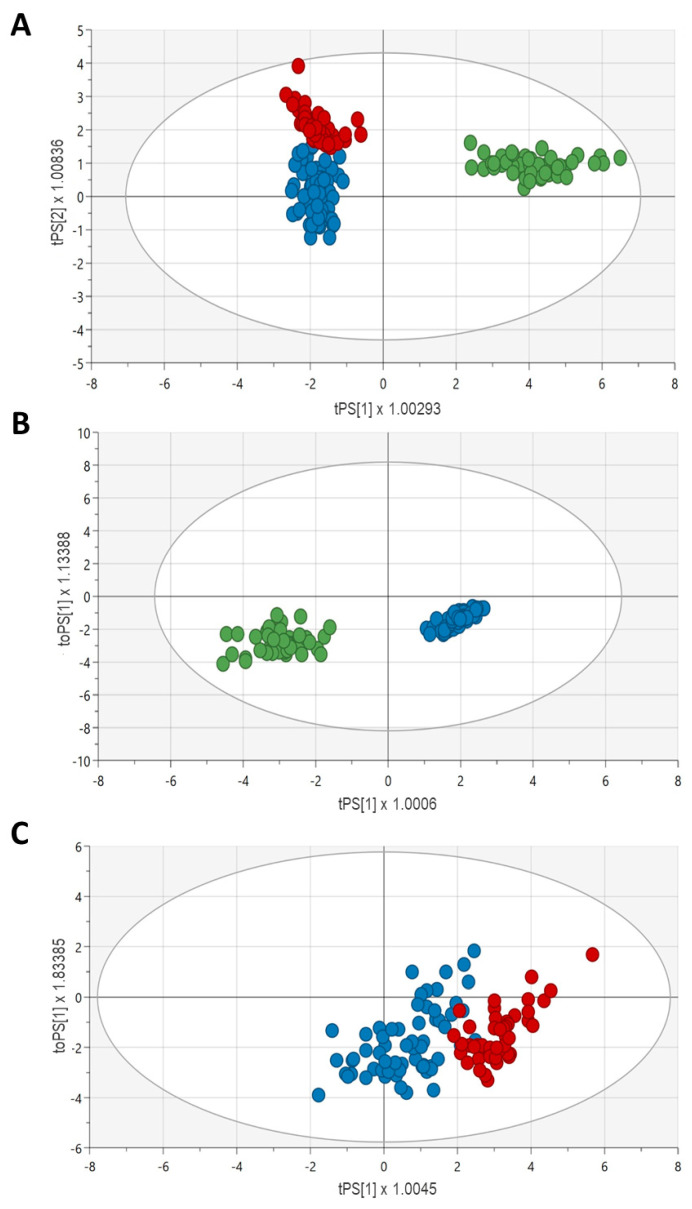
Validated OPLS-DA score plots (validated sets) for the discrimination between Controls/CKD/ESKD (**A**), Controls/CKD (**B**), and CKD/ESKD (**C**). Blue, CKD patients; red, ESKD patients; green, control subjects.

PCA analyses were performed to identify outliers who were removed before creating the OPLS-DA model. Figure 4A shows the OPLS-DA scores plot (R2X: 0.75; R2Y: 0.67; Q: 20.65, *p* < 0.0001) for the three study groups (validated sets), which revealed a good clusterization of the control samples based on the obtained metabolic profiles. However, these did not segregate CKD and ESKD patients as efficiently. The best five markers for segregation of the three groups were first glutamine, followed by D-serine, tryptophan, serotonin, and histidine (VIP scores from 1.35 to 1.26). A robust model with high predictive capability was obtained for the Controls vs. CKD comparison (R2X: 0.70; R2Y: 0.91; Q2: 0.90, *p* < 0.0001; Figure 4B). In contrast, the model for CKD vs. ESKD yielded more modest results (R2X: 0.56, R2Y: 0.47, Q2: 0.44, *p* < 0.0001; Figure 4C). The top five metabolites in decreasing order of importance for Controls/CKD were citrulline, serotonin, glutamine, GABA, and ADMA (VIP scores from 1.63 to 1.29); only citrulline and glutamine were upregulated in CKD (coefficients = 0.103 and 0.119, respectively). With regard to CKD/ESKD, tryptophan, valine, tyrosine, leucine, and histidine were the top five metabolites involved (VIP scores from 1.47 to 1.36). All of them were downregulated in the ESKD group, with tryptophan showing the highest coefficient (−0.158).

### 3.4. Amino Acids and Amines as Biomarkers of Renal Impairment

**Figure 5:**
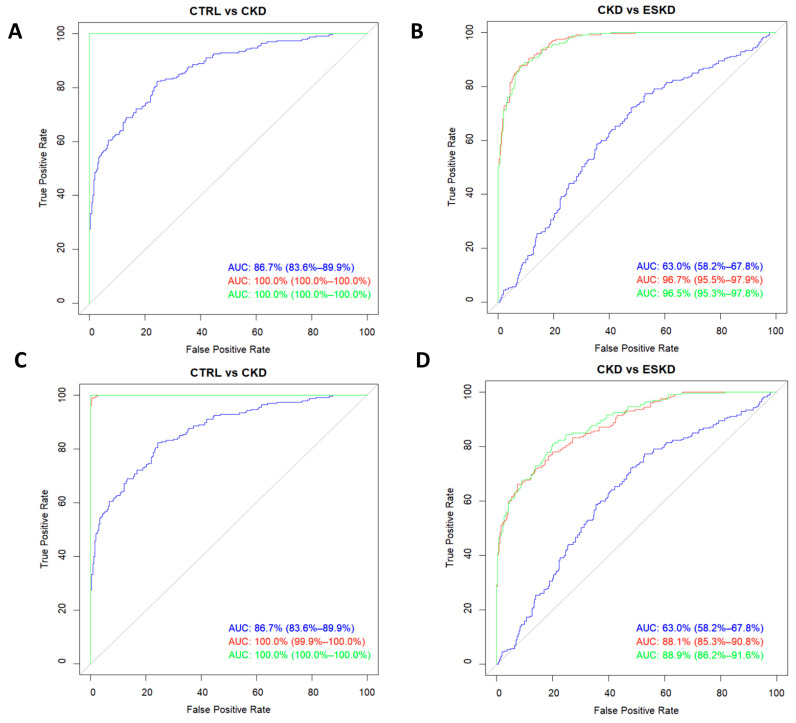
Receiver operating characteristic curves for the risk of chronic kidney disease (**A**,**C**) and the risk of progression to ESKD from CKD (**B**,**D**). Curves were modelled with the whole metabolic profile assayed (**A**,**B**) or with only the five analytes showing the highest VIP scores (**C**,**D**). The blue line corresponds to models with classic risk factors only, the green line belongs to the same model after adding the metabolic profiles, and the red line denotes the predictive ability of the metabolic profiles on their own.

Finally, we evaluated the potential role as biomarkers of renal disease of these identified relevant metabolites using ROC analysis. Figure 5A shows that the addition of the metabolic signature containing all 28 analytes to a model made up of classic risk factors for CKD (age, sex, BMI, diabetes, and hypertension) significantly increased its AUC from 86.7% (83.6–89.9) to 100% (100–100), *p* < 0.0001. The increase to maximum AUC was maintained when only the aforementioned top-five metabolites were added (Figure 5C; *p* < 0.0001). With regard to the risk of progression from CKD to ESKD, the addition of the whole metabolic profile (Figure 5B) to the classic risk model elevated the AUC from 63.0% (58.2–67.8) to 96.5% (95.3–97.8), *p* < 0.0001; however, when only the top-5 metabolites were included (Figure 5D), the increase was still significant (*p* < 0.0001) but markedly lower [final AUC = 88.9% (86.2–91.6)].

## 4. Discussion

CKD is a major public health concern for which the burden has not declined at the same rate as other important non-communicable diseases [16]. Early diagnosis of CKD would allow reducing cardiovascular complications as well as preventing fast progression to ESKD, whose patients are 100 times more likely to die of cardiovascular disease than the general population [17]. It has been suggested that in many patients a low eGFR may reflect a variety of age-related coexisting comorbid conditions and therefore be a better predictor of global health outcomes rather than CKD progression outcomes [18]. In this context, adding new biomarkers, such as AA and biogenic amines profiling, to the current evaluation of CKD could be useful to improving our existing prognostic tools.

In our study, the raw comparison of plasma levels showed great differences for all 28 compounds assayed between controls and CKD patients. The OPLS-DA models show that citrulline had the highest VIP score for the discrimination between them, being upregulated in CKD. Two large longitudinal studies, one American [19] and one Korean [10], including subjects without CKD at baseline, measured plasma metabolite concentrations during an 8-year follow-up. Both these studies also highlighted citrulline as being significantly associated with incident CKD. Citrulline metabolism occurs preferentially in proximal tubular cells [20]; therefore, renal injury could very well translate into the observed accumulation of plasma citrulline in CKD patients [12,21,22]. In this regard, it has been shown that reduced kidney function is also associated with greater citrulline excretion, suggesting that elevated citrullinemia is due to metabolic causes and not just retention [12]. This deficient citrulline biotransformation in the kidney may easily lead to decreased renal function as seen in animal models [23] since it would imply a reduction of arginine bioavailability, an AA that is necessary for endothelial nitric oxide synthesis [24].

Our findings revealed that the kynurenine/tryptophan ratio showed the strongest correlation with the eGFR. This ratio, which has also been associated with CKD in the past [10,25], reflects the activity of indoleamine dioxygenase (IDO), which is the first step of the kynurenine pathway of tryptophan metabolism. The mechanism by which kynurenine accumulation and declining tryptophan plasma concentrations (higher ratios) may be associated with reduced kidney function is discussed below in the context of ESKD. The potential of this ratio to be a good measure of early CKD risk, a pressing need in this pathology, has been previously stressed by Lee et al., who reported an OR of 12.65 (6.55–24.44) for CKD prevalence and 3.20 (1.57–6.51) for CKD incidence [10].

In general, differences in the measured metabolites between CKD and ESKD patients were less marked than those observed between controls and CKD patients. This could be partly explained by the large number of patients with CKD stage 4 in our population (*n* = 117, 50.6% of the CKD group), who display eGFR values lower than 30 mL/min/1.73 m^2^ and therefore could be more similar to ESKD patients. In any case, the addition of our metabolic profile to a predictive model based on demographic, clinical, and physiological characteristics improved its ability to discriminate CKD from ESKD by up to 96.5% with an AUC value that is in the same range as that recently reported for a similar model with 100 metabolites (99.9%) [21].

Despite the aforementioned low average eGFR in the CKD group, our findings showed 12 metabolites that were still significantly altered between ESKD and CKD. Interestingly, 10 of these, namely alanine, arginine, citrulline, glutamine, histidine, isoleucine, serine, tryptophan, valine, and ADMA, had also been shown to be able to discriminate between CKD and dialysis in a previous small study [12]. Another recent metabolomic study reported that BCAA metabolism was downregulated in ESKD compared to CKD patients [21]. We did not measure BCAA metabolites; however,, in line with this report, levels of two of the three BCAAs, valine and isoleucine, were also significantly reduced in our ESKD sample. In addition, our findings show that valine and leucine were the second and fourth most important metabolites, respectively, to discriminate CKD from ESKD according to their VIP scores. There are several explanations for this finding. BCAAs are anabolic, essential AAs, and their levels have been shown to decrease with metabolic acidosis, which worsens with CKD progression to ESKD because of enhanced proteolysis and BCAA catabolism [26]. Moreover, the levels of BCAAs and other essential AAs have also been shown to be lower in ESKD patients due to hemodialysis and reduced protein intake [27].

The most relevant metabolite for CKD/ESKD discrimination in our study was tryptophan, which showed the highest VIP score and the highest negative coefficient in the OPLS-DA model. Tryptophan catabolism by IDO leading to kynurenine is known to increase with CKD progression and dialysis [28,29]. The upregulation of this pathway is supported by our aforementioned observation that the kynurenine/tryptophan ratio displayed an inverse high correlation with eGFR. In the same line, two other metabolomic studies, although with only 32 and 77 patients [12,21], have also reported the increase in similar ratios (using different metabolites in the kynurenine pathway) in ESKD patients. Kynurenine is further biotransformed into several metabolites that play a relevant role in chronic inflammation, oxidative stress, and apoptosis [30,31], which could explain the increased inflammatory response and cell damage present in advanced CKD. Indeed, modification of the kynurenine pathway via inhibition of kynurenine hydroxylase has been shown to attenuate surgical complications in rodents [32], which suggests that the downregulation of tryptophan catabolism to kynurenine might be a promising therapy to delay progression to ESKD.

This study has a number of strengths and limitations. A clear strong point was the sample size, which was far higher than that reported by the vast majority of studies analyzing biological concentrations of AAs in renal patients, and that allowed capture of the true variability of the population in terms of the concentrations of these compounds. Among the limitations, urine samples were not available for the control group, and, hence, proteinuria values could not be obtained for these subjects. In the same manner, protein intake of participants was not assessed. However, even though a higher risk of malnutrition was observed for patients with advanced CKD (significantly lower Val/Gly and EAA/NEAA ratios), multivariate models showed how eGFR was a far better predictor of AA plasma concentrations than albumin values, indicating that nutrition status by itself would not explain our findings. Another limitation was the lack of a validation cohort for the reported observations.

In summary, we have shown how in a large population of healthy subjects and renal patients with a wide range of eGFR values, the use of a LC/MS-based targeted metabolomics approach may be useful to identify prognostic biomarkers of CKD risk and progression. Our results taken together indicate that plasma concentrations of AA and related amines have a high degree of predictive ability regarding renal function. In particular, citrulline and the tryptophan metabolic route to kynurenine showed the greatest potential as biomarkers, and the addition of these factors to the current evaluation of CDK risk and progression might improve the accuracy of the existing prognostic methods largely based on creatinine alone.

## Figures and Tables

**Table 1:** Demographic and clinical characteristics of the population of study stratified by renal status.

	CTRL (*n* = 264)		CKD (*n* = 231)			ESKD (*n* = 325)		
	Mean/*n*	SD/%	Mean/*n*	SD/%	p^a^	Mean/*n*	SD/%	p^b^
Age	75.92	7.10	65.91	12.83	<0.001	68.79	13.20	0.004
Sex					<0.001			
Men	125	47.3%	151	65.4%		205	63.1%	0.321
Women	139	52.7%	80	34.6%		120	36.9%	
BMI	27.64	4.01	30.14	5.54	<0.001	28.44	5.89	<0.001
Serum creatinine (g/dL)	0.93	0.25	2.09	0.91	<0.001	5.50	1.93	<0.001
eGFR (mL/min/1.73 m^2^)	71.47		40.32		<0.001	11.36		<0.001
Proteins, 24 h	Na		839.17	1404.27		1989.02	2053.95	<0.001
Albumin, 24 h	Na		574.87	1107.30		1387.46	1460.12	<0.001
Creatinine, 24 h	Na		1267.56	1072.39		863.01	350.54	<0.001
ACR	Na		520.14	951.91		1634.86	1601.60	<0.001
Calcium (mg/dL)	9.47	0.36	9.73	4.21	0.913	9.28	5.61	<0.001
Phosphorus (mg/dL)	3.25	0.50	3.51	0.66	<0.001	4.35	1.12	<0.001
PTH (pg/mL)	66.84	31.94	173.88	143.61	<0.001	333.64	244.53	<0.001
DM								
No	220	0.83	114	0.49	<0.001	152	47.6%	0.379
Yes	44	0.17	117	0.51		167	52.4%	
Hypertension								
No	139	52.7%	48	20.8%	<0.001	68	21.3%	0.483
Yes	125	47.3%	183	79.2%		251	78.7%	
Hyperlipidemia								
No	174	65.9%	129	55.8%	0.014	146	45.2%	0.009
Yes	90	34.1%	102	44.2%		177	54.8%	
Smoking								
Nonsmoker	188	72.9%	102	44.2%	<0.001	166	51.9%	0.002
Smoker	14	5.4%	52	22.5%		36	11.3%	
Former smoker	56	21.7%	77	33.3%		118	36.9%	

CTRL, control group; CKD, chronic kidney disease; ESKD, end-stage kidney disease; BMI, body-mass index; Na, not available; ACR, albumin-to-creatinine ratio. p^a^, comparisons between control and CKD groups; p^b^, comparisons between CKD and ESKD groups.

**Table 2:** Plasma concentrations (mmol/L) of the amino acids and amines assayed. CTRL, control subjects; CKD, chronic kidney disease patients; ESKD, end-stage kidney disease patients.

	CTRL		CKD		ESKD		*p*-Value
	Mean	SD	Mean	SD	Mean	SD	CKD/ESKD
Alanine	527.24	397.88	279.59	112.52	150.17	88.45	<0.0001
Arginine	96.28	73.09	58.40	19.49	41.51	20.77	<0.0001
Asparagine	23.34	27.69	1.23	0.55	0.77	0.31	1.000
Aspartate	32.90	50.38	3.18	1.96	2.12	1.05	1.000
Citrulline	27.33	11.49	93.45	42.29	70.09	48.87	<0.0001
Glycine	49.60	56.69	101.82	58.36	101.62	78.18	1.000
Glutamate	150.35	133.99	57.01	36.94	37.73	23.87	0.016
Glutamine	226.53	123.81	769.87	393.51	415.60	254.99	<0.0001
Histidine	71.72	59.13	108.59	42.72	54.26	34.46	<0.0001
Isoleucine	110.40	131.03	74.14	29.25	50.62	19.27	0.001
Leucine	743.67	989.98	86.89	30.38	54.23	19.26	1.000
Lysine	134.94	165.88	50.36	24.83	30.67	16.16	0.051
Methionine	11.15	19.13	6.05	3.75	3.52	6.93	0.041
Phenylalanine	318.80	1232.85	60.20	19.32	43.20	19.31	1.000
Proline	301.09	266.91	238.12	107.00	194.28	225.01	0.054
L-Serine	59.21	61.87	2.81	3.02	1.63	1.38	1.000
Tyrosine	527.33	718.83	54.80	29.97	23.05	16.99	1.000
Threonine	240.18	226.74	52.20	13.80	40.39	13.11	0.865
Tryptophan	139.90	65.27	80.57	35.99	40.62	13.71	<0.0001
Valine	462.07	482.30	268.64	135.06	125.82	78.09	<0.0001
Acetylcholine	0.76	0.43	1.15	0.42	1.15	0.49	1.000
D-Serine	0.66	0.35	0.29	0.30	0.70	0.29	<0.0001
Kynurenine	1.98	0.78	2.44	1.20	3.38	1.39	<0.0001
Kynurenic acid	1.96	0.81	1.52	1.01	1.03	0.19	<0.0001
ADMA	0.84	0.33	0.38	0.19	0.26	0.19	<0.0001
Creatine	20.52	10.92	11.91	9.40	9.03	10.62	0.004
GABA	0.22	0.09	0.05	0.11	0.04	0.03	0.739
Serotonin	0.63	0.25	0.16	0.17	0.14	0.03	0.762

SD, standard deviation.

## Data Availability

Source data for this study are openly available at DOI 10.6084/m9.figshare.23617200.

## Supplementary Materials

The following supporting information can be downloaded at: https://www.mdpi.com/article/10.3390/biomedicines11102775/s1, Table S1: Calibration standards; Table S2: Multiple reaction monitoring conditions for the metabolites; Table S3: Multivariate regression parameters for the effect of renal status (control, chronic kidney disease, or end-stage kidney disease) on the metabolites’ plasma levels. Models were adjusted for age, sex, body-mass index, hypertension, smoking status, and diabetes; Figure S1: Correlation of amino acid ratios with the estimated glomerular filtration rate in patients with different degrees of CKD and healthy volunteers.

## References

[1] Foreman K.J., Marquez N., Dolgert A., Fukutaki K., Fullman N., McGaughey M., Pletcher M.A., Smith A.E., Tang K., Yuan C.W. (2018). Forecasting life expectancy, years of life lost, and all-cause and cause-specific mortality for 250 causes of death: Reference and alternative scenarios for 2016–40 for 195 countries and territories. Lancet.

[2] Ortiz A. (2022). RICORS2040: The need for collaborative research in chronic kidney disease. Clin. Kidney J..

[3] Winearls C.G., Haynes R., Glassock R. (2010). CKD staging—Evolution not revolution. Nefrol. Engl. Ed..

[4] Garibotto G., Sofia A., Saffioti S., Bonanni A., Mannucci I., Verzola D. (2010). Amino acid and protein metabolism in the human kidney and in patients with chronic kidney disease. Clin. Nutr..

[5] Le A., Ng A., Kwan T., Cusmano-Ozog K., Cowan T.M. (2014). A rapid, sensitive method for quantitative analysis of underivatized amino acids by liquid chromatography-tandem mass spectrometry (LC-MS/MS). J. Chromatogr. B Analyt. Technol. Biomed. Life Sci..

[6] Merchant M.L. (2010). Mass spectrometry in chronic kidney disease research. Adv. Chronic Kidney Dis..

[7] Fenn J.B., Mann M., Meng C.K., Wong S.F., Whitehouse C.M. (1989). Electrospray ionization for mass spectrometry of large biomolecules. Science.

[8] Yates J.R., Link A.J., Schieltz D. (2000). Direct analysis of proteins in mixtures. Application to protein complexes. Methods Mol. Biol..

[9] Benito S., Sanchez-Ortega A., Unceta N., Goicolea M.A., Barrio R.J. (2019). LC-QQQ-MS routine analysis method for new biomarker quantification in plasma aimed at early chronic kidney disease diagnosis. J. Pharm. Biomed. Anal..

[10] Lee H., Jang H.B., Yoo M.G., Park S.I., Lee H.J. (2020). Amino Acid Metabolites Associated with Chronic Kidney Disease: An Eight-Year Follow-Up Korean Epidemiology Study. Biomedicines.

[11] Silva R.E., Baldim J.L., Chagas-Paula D.A., Soares M.G., Lago J.H.G., Goncalves R.V., Novaes R.D. (2018). Predictive metabolomic signatures of end-stage renal disease: A multivariate analysis of population-based data. Biochimie.

[12] Duranton F., Lundin U., Gayrard N., Mischak H., Aparicio M., Mourad G., Daures J.P., Weinberger K.M., Argiles A. (2014). Plasma and urinary amino acid metabolomic profiling in patients with different levels of kidney function. Clin. J. Am. Soc. Nephrol. CJASN.

[13] Ghanem S.E., Abdel-Samiee M., El-Said H., Youssef M.I., ElZohry H.A., Abdelsameea E., Moaz I., Abdelwahab S.F., Elaskary S.A., Zaher E.M. (2022). Evaluation of Amino Acids Profile as Non-Invasive Biomarkers of Hepatocellular Carcinoma in Egyptians. Trop. Med. Infect. Dis..

[14] Leonetti S., Herzog R.I., Caprio S., Santoro N., Trico D. (2020). Glutamate-Serine-Glycine Index: A Novel Potential Biomarker in Pediatric Non-Alcoholic Fatty Liver Disease. Children.

[15] Wong J.M., Malec P.A., Mabrouk O.S., Ro J., Dus M., Kennedy R.T. (2016). Benzoyl chloride derivatization with liquid chromatography-mass spectrometry for targeted metabolomics of neurochemicals in biological samples. J. Chromatogr. A.

[16] Xie Y., Bowe B., Mokdad A.H., Xian H., Yan Y., Li T., Maddukuri G., Tsai C.Y., Floyd T., Al-Aly Z. (2018). Analysis of the Global Burden of Disease study highlights the global, regional, and national trends of chronic kidney disease epidemiology from 1990 to 2016. Kidney Int..

[17] Baigent C., Burbury K., Wheeler D. (2000). Premature cardiovascular disease in chronic renal failure. Lancet.

[18] O’Hare A.M., Bertenthal D., Walter L.C., Garg A.X., Covinsky K., Kaufman J.S., Rodriguez R.A., Allon M. (2007). When to refer patients with chronic kidney disease for vascular access surgery: Should age be a consideration?. Kidney Int..

[19] Rhee E.P., Clish C.B., Ghorbani A., Larson M.G., Elmariah S., McCabe E., Yang Q., Cheng S., Pierce K., Deik A. (2013). A combined epidemiologic and metabolomic approach improves CKD prediction. J. Am. Soc. Nephrol..

[20] Levillain O., Hus-Citharel A., Morel F., Bankir L. (1990). Localization of arginine synthesis along rat nephron. Am. J. Physiol..

[21] Dahabiyeh L.A., Nimer R.M., Sumaily K.M., Alabdaljabar M.S., Jacob M., Sabi E.M., Hussein M.H., Abdel Rahman A. (2023). Metabolomics profiling distinctively identified end-stage renal disease patients from chronic kidney disease patients. Sci. Rep..

[22] Shah V.O., Townsend R.R., Feldman H.I., Pappan K.L., Kensicki E., Vander Jagt D.L. (2013). Plasma metabolomic profiles in different stages of CKD. Clin. J. Am. Soc. Nephrol..

[23] Al Banchaabouchi M., Marescau B., D’Hooge R., Engelborghs S., De Deyn P.P. (2000). Consequences of renal mass reduction on amino acid and biogenic amine levels in nephrectomized mice. Amino Acids.

[24] Shimabukuro M. (2023). L-Arginine, Nitric Oxide, and Endothelial Dysfunction Underlying Atherosclerotic Cardiovascular Disease (ASCVD). J. Atheroscler. Thromb..

[25] Goek O.N., Prehn C., Sekula P., Romisch-Margl W., Doring A., Gieger C., Heier M., Koenig W., Wang-Sattler R., Illig T. (2013). Metabolites associate with kidney function decline and incident chronic kidney disease in the general population. Nephrol. Dial. Transplant..

[26] Hara Y., May R.C., Kelly R.A., Mitch W.E. (1987). Acidosis, not azotemia, stimulates branched-chain, amino acid catabolism in uremic rats. Kidney Int..

[27] Holecek M. (2018). Branched-chain amino acids in health and disease: Metabolism, alterations in blood plasma, and as supplements. Nutr. Metab..

[28] Koenig P., Nagl C., Neurauter G., Schennach H., Brandacher G., Fuchs D. (2010). Enhanced degradation of tryptophan in patients on hemodialysis. Clin. Nephrol..

[29] Mor A., Kalaska B., Pawlak D. (2020). Kynurenine Pathway in Chronic Kidney Disease: What’s Old, What’s New, and What’s Next?. Int. J. Tryptophan Res..

[30] Gonzalez Esquivel D., Ramirez-Ortega D., Pineda B., Castro N., Rios C., Perez de la Cruz V. (2017). Kynurenine pathway metabolites and enzymes involved in redox reactions. Neuropharmacology.

[31] Zhang Q., Sun Y., He Z., Xu Y., Li X., Ding J., Lu M., Hu G. (2020). Kynurenine regulates NLRP2 inflammasome in astrocytes and its implications in depression. Brain. Behav. Immun..

[32] Zakhary G., Sherchan P., Li Q., Tang J., Zhang J.H. (2020). Modification of kynurenine pathway via inhibition of kynurenine hydroxylase attenuates surgical brain injury complications in a male rat model. J. Neurosci. Res..

