# Supplementary material for: Prognostic Significance of Amino Acid and Biogenic Amines Profiling in Chronic Kidney Disease
Source: Biomedicines. 2023 Oct 13;11(10):2775. doi: 10.3390/biomedicines11102775 (PMC10604890; doi:10.3390/biomedicines11102775)
Supplement: Supplementary file 1 [file biomedicines-11-02775-s001.zip › REVISED_Supplementary tables.pdf]

**Supplementary Table S1**. Calibration standards.

| ANALYTE | Dynamic range of the curve (ng/ml) |
|---|---|
| Acetylcholine | 3,12-400 |
| Alanine | 173,4-88800 |
| Arginine | 679,7-87000 |
| Asparagine | 62,5-20000 |
| Aspartate | 259,37-66400 |
| Asymmetric dimethylarginine (ADMA) | 7,81-500 |
| Citrulline | 234,37-60000 |
| Creatine | 62,5-8000 |
| Gamma-aminobutyric acid (GABA) | 3,12-200 |
| Glutamate | 286,7-146800 |
| Glutamine | 15625-250000 |
| Glycine | 292,2-74800 |
| Histidine | 292,96-154800 |
| Isoleucine | 514-131600 |
| Kynurenic acid | 187,5-6000 |
| Kynurenine | 9,76-5000 |
| Leucine | 515,6-132000 |
| Lysine | 285,15-146000 |
| Methionine | 3,66-30000 |
| Phenylalanine | 1287,5-82400 |
| Proline | 224,2-114800 |
| D-Serine | 117,18-1875 |
| L-Serine | 117,18-1875 |
| Serotonin | 4,68-600 |
| Threonine | 232-118800 |
| Tryptophan | 5625-180000 |
| Tyrosine | 706,25-90400 |
| Valine | 457,8-117200 |

**Supplementary Table S2**. Multiple reaction monitoring conditions for the metabolites.

| | COMPOUND NAME | TRANSITION (M/Z) | FRAGMENTOR (V) | COLLISION ENERGY (V) | RETENTION TIME (MIN) |
|---|---|---|---|---|---|
| 1 | Acetylcholine | 146-87 | 120 | 15 | 2.15 |
| | D4-Acetylcholine | 150-91 | 120 | 15 | 2.15 |
| 2 | Alanine | 194-105 | 120 | 20 | 10.08 |
| | 13C6Bz-Alanine | 200-111 | 120 | 20 | 10.08 |
| 3 | Arginine | 279-105 | 135 | 30 | 7.68 |
| | 13C6Bz-Arginine | 285-111 | 135 | 30 | 7.68 |
| 4 | Asparagine | 237-105 | 120 | 20 | 7.46 |
| | 13C6Bz-Asparagine | 243-111 | 120 | 20 | 7.46 |
| 5 | Aspartate | 238-105 | 120 | 10 | 8.46 |
| | 13C6Bz-Aspartate | 244-111 | 120 | 10 | 8.46 |
| 6 | Asymmetric dimethylarginine (ADMA) | 307.2-105 | 70 | 20 | 8.04 |
| | 13C6Bz- Asymmetric dimethylarginine (ADMA) | 313-111 | 70 | 20 | 8.04 |
| 7 | Citrulline | 280-105 | 120 | 20 | 8.28 |
| | 13C6Bz-Citrulline | 286-111 | 120 | 20 | 8.28 |
| 8 | Creatine | 132.1-90.2 | 90 | 9 | 2 |
| 9 | Gamma-aminobutyric acid (GABA) | 208-105 | 120 | 10 | 10.36 |
| | 13C6Bz-Gamma-aminobutyric acid (GABA) | 214-111 | 120 | 10 | 10.36 |
| 10 | Glutamate | 252-105 | 120 | 20 | 9.08 |
| | 13C6Bz-Glutamate | 258-111 | 120 | 20 | 9.08 |
| 11 | Glutamine | 251-105 | 120 | 20 | 8.17 |
| | 13C6Bz-Glutamine | 257-111 | 120 | 20 | 8.17 |
| 12 | Glycine | 180-105 | 120 | 10 | 8.79 |
| | 13C6Bz-Glycine | 186-111 | 120 | 10 | 8.79 |
| 13 | Histidine | 260-110 | 130 | 20 | 6.9 |
| | 13C6Bz-Histidine | 266-110 | 130 | 20 | 6.9 |
| 14 | Isoleucine | 236-105 | 120 | 30 | 15.9 |
| | 13C6Bz-Isoleucine | 242-111 | 120 | 30 | 15.9 |
| 15 | Kynurenic acid | 294-105 | 120 | 30 | 18.5 |
| | 13C6Bz-Kynurenic acid | 300-111 | 120 | 30 | 18.5 |
| 16 | Kynurenine | 417-122 | 120 | 10 | 19.02 |
| | 13C6Bz-Kynurenine | 429-128 | 120 | 10 | 19.02 |
| 17 | Leucine | 236-105 | 120 | 30 | 16.31 |
| | 13C6Bz-Leucine | 242-111 | 120 | 30 | 16.31 |
| 18 | Lysine | 355-188 | 120 | 20 | 14.7 |
| | 13C6Bz-Lysine | 367-194 | 120 | 20 | 14.7 |
| 19 | Methionine | 254-105 | 120 | 15 | 13.69 |
| | 13C6Bz-Methionine | 260-111 | 120 | 15 | 13.69 |
| 20 | Phenylalanine | 270-120 | 120 | 10 | 16.48 |
| | 13C6Bz-Phenylalanine | 276-120 | 120 | 10 | 16.48 |
| 21 | Proline | 220-105 | 120 | 20 | 11.03 |
| | 13C6Bz-Proline | 226-111 | 120 | 20 | 11.03 |
| 22 | D-Serine | 210-105 | 120 | 20 | 7.70 |
| | 13C6Bz-D-Serine | 216-111 | 120 | 20 | 7.70 |
| 23 | L-Serine | 210-105 | 120 | 20 | 7.99 |
| | 13C6Bz-L-Serine | 216-111 | 120 | 20 | 7.99 |
| 24 | Serotonine | 385-264 | 140 | 20 | 20.6 |
| | 13C6Bz-Serotonine | 397-270 | 140 | 20 | 20.6 |
| 25 | Threonine | 224-105 | 140 | 20 | 16.5 |
| | 13C6Bz-Threonine | 230-111 | 140 | 20 | 16.5 |
| 26 | Tryptophan | 309-159 | 120 | 10 | 16.54 |
| | 13C6Bz-Tryptophan | 315-159 | 120 | 10 | 16.54 |
| 27 | Tyrosine | 390-105 | 120 | 30 | 19.67 |
| | 13C6Bz-Tyrosine | 402-111 | 120 | 30 | 19.67 |
| 28 | Valine | 222-105 | 120 | 30 | 13.61 |
| | 13C6Bz-Valine | 228-111 | 120 | 30 | 13.61 |

**Supplementary Table S3.** Multivariate regression parameters for the effect of renal status (control, chronic kidney disease or end-stage kidney disease) on the metabolites' plasma levels. Models were adjusted for age, sex, body-mass index, hypertension, smoking status and diabetes.

| | B | 95%CI | SE | Beta | p-value |
|---|---|---|---|---|---|
| Alanine | -190.15 | (-212.95 - -167.28) | 11.63 | -0.547 | <0.0001 |
| Arginine | -27.45 | (-31.72 - -23.18) | 2.18 | -0.451 | <0.0001 |
| Asparagine | -9.9 | (-11.46 - -8.35) | 0.79 | -0.434 | <0.0001 |
| Aspartate | -13.84 | (-16.63 - -11.08) | 1.42 | -0.358 | <0.0001 |
| Citrulline | 13.41 | (9.4 - 17.42) | 2.04 | 0.242 | <0.0001 |
| Glycine | 22.12 | (15.85 - 28.39) | 3.19 | 0.267 | <0.0001 |
| Glutamate | -57.84 | (-65.62 - -50.06) | 3.96 | -0.509 | <0.0001 |
| Glutamine | 30.11 | (-1.12 - 61.35) | 15.91 | 0.073 | 0.059 |
| Histidine | -17.38 | (-22.02 - -12.73) | 2.37 | -0.283 | <0.0001 |
| Isoleucine | -31.76 | (-39.12 - -24.41) | 3.75 | -0.353 | <0.0001 |
| Leucine | -301.77 | (-356.75 - -246.79) | 28.01 | -0.387 | <0.0001 |
| Lysine | -47.26 | (-56.45 - -38.07) | 4.68 | -0.369 | <0.0001 |
| Methionine | -3.97 | (-5.11 - -2.83) | 0.58 | -0.267 | <0.0001 |
| Phenylalanine | -118.01 | (-185.38 - -50.64) | 34.32 | -0.137 | 0.001 |
| Proline | -58.57 | (-79.01 - -38.12) | 10.42 | -0.221 | <0.0001 |
| D-Serine | 0.07 | (0.03 - 0.1) | 0.02 | 0.152 | <0.0001 |
| L-Serine | -24.6 | (-28.09 - -21.11) | 1.78 | -0.461 | <0.0001 |
| Tyrosine | -215.74 | (-255.46 - -176.02) | 20.23 | -0.381 | <0.0001 |
| Threonine | -87.45 | (-100.22 - -74.69) | 6.5 | -0.458 | <0.0001 |
| Tryptophan | -49.48 | (-53.56 - -45.42) | 2.07 | -0.689 | <0.0001 |
| Valine | -175.51 | (-202.94 - -148.09) | 13.97 | -0.454 | <0.0001 |
| Acetylcholine | 0.16 | (0.12 - 1) | 0.02 | 0.271 | <0.0001 |
| Kynurenic acid | -0.49 | (-0.56 - -0.42) | 0.04 | -0.498 | <0.0001 |
| ADMA | -0.29 | (-0.32 - -0.27) | 0.01 | -0.693 | <0.0001 |
| Creatine | -5.9 | (-6.83 - -4.96) | 0.48 | -0.435 | <0.0001 |
| GABA | -0.08 | (-0.09 - -0.08) | 0.004 | -0.617 | <0.0001 |
| Kynurenine | -0.02 | (-0.13 - 0.1) | 0.06 | -0.012 | 0.755 |
| Serotonin | -0.22 | (-0.24 - -0.2) | 0.01 | -0.663 | <0.0001 |

B, regression coefficient; 95%CI, 95% confidence intervals; SE, standard error; Beta, standardized coefficient
