# Supplementary figures and images for: Prognostic Significance of Amino Acid and Biogenic Amines Profiling in Chronic Kidney Disease
Source: Biomedicines. 2023 Oct 13;11(10):2775. doi: 10.3390/biomedicines11102775 (PMC10604890; doi:10.3390/biomedicines11102775)

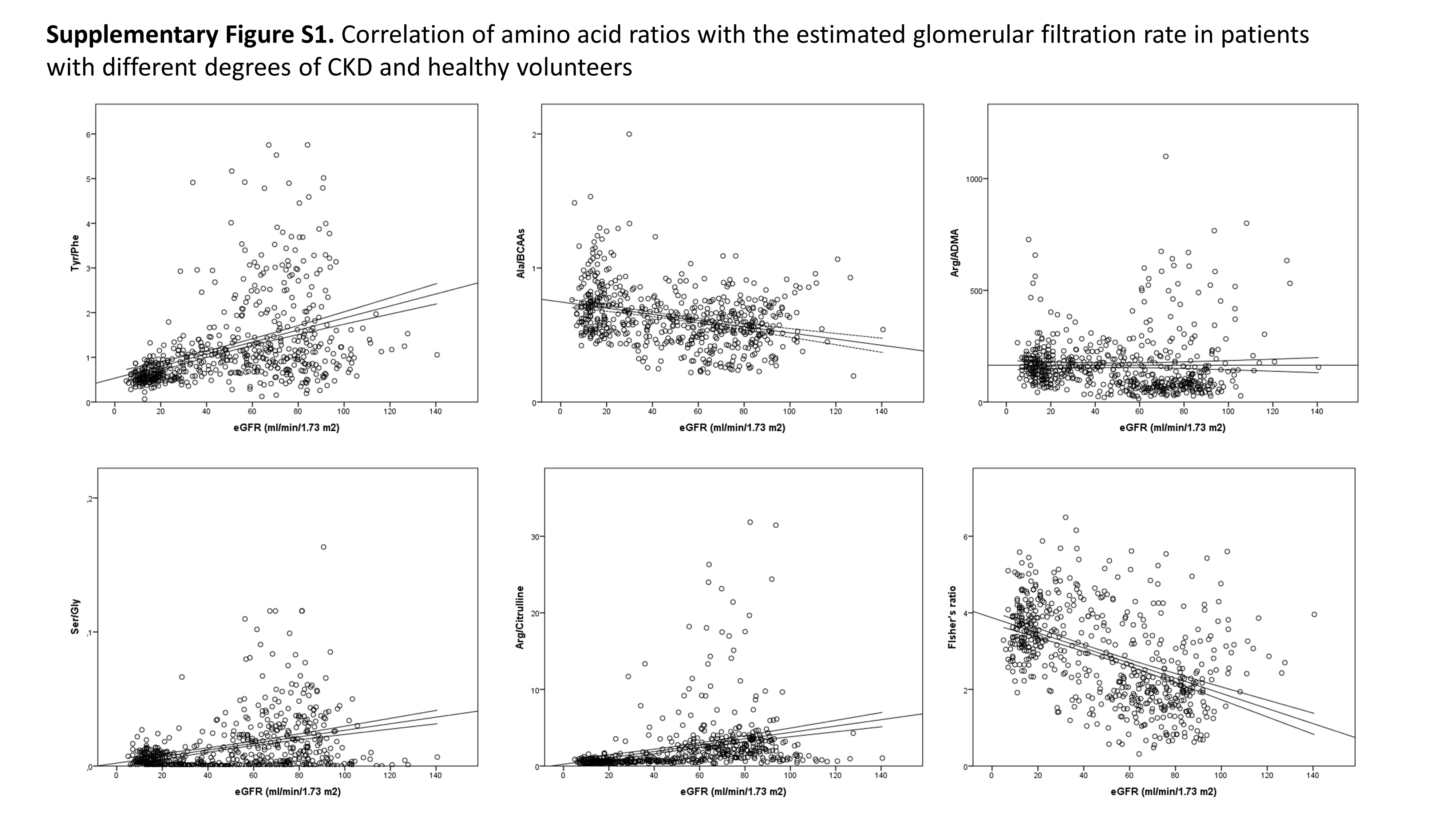

Supplement: Supplementary file 1 [file biomedicines-11-02775-s001.zip › Suppl Fig S1.TIF]
